# Butorphanol as an Adjuvant to Ropivacaine for Adductor Canal Blocks in Total Knee Arthroplasty Patients: A Randomized, Double, Blind Study

**DOI:** 10.1155/2022/7718108

**Published:** 2022-10-14

**Authors:** Tong Mu, Danyan Liu, Fei Gao

**Affiliations:** Department of Anesthesiology, The First Affiliated Hospital of Chongqing Medical University, Chongqing, China

## Abstract

**Background:**

The objective of this study was to observe the effects of butorphanol as an adjuvant to ropivacaine for the adductor canal block (ACB) on postoperative analgesia in patients undergoing total knee arthroplasty (TKA).

**Methods:**

Seventy-four patients undergoing TKA were included and randomly divided into two groups: Group BR received 20 ml of 0.33% ropivacaine plus 1 mg butorphanol and Group R received 20 ml of 0.33% ropivacaine plus 1 ml normal saline for ultrasound-guided adductor canal blocks. The primary outcomes were the duration of the sensory block and the pain visual analogue scale (VAS), and secondary outcomes included the number of PCIA attempts (patient-controlled intravenous analgesia) and the time to first pressing and rescue analgesia. Other outcomes included knee active range of motion (ROM), quadriceps strength, the time to first mobilization, the duration of postoperative hospital stay, Knee Society Score (KSS), and postoperative complications.

**Results:**

Since two patients in each group rejected postoperative assessments, 35 patients were included in each group. Compared with Group R, Group BR had longer duration of sensory blocks (18.42 ± 3.46 vs. 15.36 ± 2.29 h, *p* < 0.01) and lower postoperative pain scores within 24 hours at rest and within 12 hours with activity (*p* < 0.01). The number of PCIA attempts decreased within 48 hours after surgery (4.5 ± 1.2 vs. 7.8 ± 1.5 times, *p* < 0.01), and the time to first pressing was later (20.31 ± 2.59 vs. 16.25 ± 2.31 h, *p* < 0.01). In addition, Group BR had bigger knee ROM at within 24 hours after the operation than Group R (68.37 ± 4.70°vs. 59.21 ± 6.41,85.67 ± 5.17 vs. 74.37 ± 4.68°, 97.62 ± 5.43 vs. 84.18 ± 4.49°, *p* < 0.01). There was no significant difference between the two groups (*p* > 0.05) in terms of rescue analgesia, quadriceps strength, the time to first mobilization, the duration of postoperative hospital stay, the KSS function scores, and postoperative complications.

**Conclusions:**

Butorphanol plus ropivacaine ultrasound-guided adductor canal block can prolong the duration of sensory block, relieve early postoperative pain, and improve the range of motion of the knee joint, without affecting the occurrence of postoperative complications. *Name of the Registry*. Chinese Clinical Trial Registry. *Trial Registration Number*. ChiCTR2100041859. *URL of Trial Registry Record*. http://www.chictr.org.cn/edit.aspx?pid=119731&htm=4. *Date of Registration*. 08/01/2021 0:00:00.

## 1. Introduction

With the aging process of society, surgical treatment has become more and more common in the elderly with degenerative knee diseases. Total knee arthroplasty (TKA), as the most effective surgical method for the treatment of end-stage knee osteoarthritis, is expected to reach 3.5 million cases in the United States by 2030 [1]. However, TKA is a highly painful operation, and nearly 80% of patients will have moderate, severe, or extreme pain after operation [2]. Insufficient postoperative analgesia will affect the early rehabilitation training of patients, increase the incidence of perioperative joint stiffness and deep venous thrombosis, and increase the hospital stay time [3]. Therefore, optimal anesthesia and analgesic methods are vital for patients undergoing TKA.

As the further development of enhanced recovery after surgery (ERAS), postoperative pain management is increasingly important [4]. There are many effective methods to relieve postoperative pain, including opioids consumption, epidural analgesia, peripheral nerve block, and periarticular infiltration analgesia [5, 6]. In recent years, the peripheral nerve block has become the best choice for postoperative analgesia of TKA. Compared with femoral nerve blocks (FNBs), an adductor canal block (ACB) can not only provide satisfactory analgesic effects but also has little effects on the muscle strength of the quadriceps femoris by blocking the saphenous nerve mainly [6, 7]. Nonetheless, the duration of single ACB analgesia is limited, and continuous ACB has the risk of catheter displacement, dislodgement, and postoperative falls [8]. Therefore, it can be a choice to prolong the analgesic time of the nerve block and strengthen the analgesic effect through local anesthetic adjuvants in single ACB.

Various adjuvants have been reported to be effective to improve the quality of block such as tramadol, buprenorphine, *α*^2^-adrenergic agonists, and dexamethasone [9], but it is unclear that which drug is the best adjuvant for local anesthetics. As a mixed agonist-antagonist to opioid receptors, the advantage of butorphanol is that it mainly stimulates *κ* receptors and has an agonistic effect and antagonistic effect on *u* receptors which cause a variety of adverse reactions. This characteristic enables it to provide effective analgesia and less adverse opioid reactions when used as an adjuvant for local anesthetics. In previous studies, butorphanol plus ropivacaine can significantly enhance the analgesic effect in upper limb peripheral nerve blocks and epidural anesthesia with less adverse reactions [10, 11]. But there is no clinical report of butorphanol combined with local anesthesia for ACB. Accordingly, the purpose of this study was to investigate the effect of adding butorphanol to ropivacaine ACB on postoperative analgesia in patients undergoing total knee arthroplasty.

## 2. Methods

### 2.1. Patients and Study Design

Patients undergoing elective first-time unilateral knee replacement surgery from April 2021 to October 2021 at the First Affiliated Hospital of Chongqing Medical University were included. This randomized controlled trial (RCT) was registered with the clinical trials registry on January 8, 2021 (identifier: ChiCTR2100041859) and approved by the clinical medical research ethics committee of the First Affiliated Hospital of Chongqing Medical University (NO. 2020-633). All participants had signed informed consent before the surgery.

The inclusion criteria were as follows: male and female aged 50–80 years were recruited with the American Society of Anesthesiologists (ASA) score II or III and had a body mass index (BMI) of 18–30 kg/m^2^. Exclusion criteria were as follows: knee deformity, flexion deformity ≥30°, varus-valgus deformity ≥30°, history of local anesthetic allergy, local skin infection by puncture, severe heart, lung, liver, kidney, and other organ diseases, coagulation dysfunction, cognitive dysfunction, and history of mental illness. Those who failed the nerve block or requested to withdraw for their own reasons were also excluded.

### 2.2. Randomization and Blinding

All patients scheduled for elective TKA were randomized into one of the two groups (B vs. BR) using a computer-generated randomization sequence. The investigator (XX) sealed the random number in opaque envelopes, and the patient selected an envelope before anesthesia to determine the treatment group. Anesthetics were prepared by the investigator (XX) who did not take part in surgery, anesthesia, outcome collection, and statistical analysis. ACBs were all performed by the same anesthesiologist who specialized in ultrasound-guided regional anesthesia. Patients, anesthetists, surgeons, nurses, data collectors, and statistical analysts were also unaware of group allocations.

### 2.3. Preoperative Management

The data collector documented preoperatively basic characteristics of patients, including gender, age, BMI, ASA degree, pain score, knee ROM, and quadriceps strength. After hospital admission, parecoxib (40 mg twice a day) was administered intravenously to control pain.

The prostheses used included Depuy P. F. C and Link. All TKAs were performed by the same team of orthopaedic surgeons using a medial patellar approach, with pneumatic tourniquets and controlled blood pressure measures during the operation.

### 2.4. Anesthesia and ACB

Each patient fasted for 8 hours and was forbidden to drink for 2 hours before surgery. In the operating room, intravenous access was secured and the basic vital signs including electrocardiogram (ECG), blood oxygen saturation (SpO_2_), invasive blood pressure (IBP), and partial pressure of end respiratory carbon dioxide (P_ET_CO_2_) were monitored. Induction of anesthesia: intravenous injection of midazolam 0.04 mg/kg, propofol 1-2 mg/kg, sufentanil 0.3–0.5 *μ*g/kg, and vecuronium 0.08–0.1 mg/kg was administered. After muscle relaxation was completely effective, a suitable tracheal tube was inserted, connected to an anesthesia machine for volume-controlled ventilation, and P_ET_CO_2_ was maintained at 35–45 mmHg. In all patients, 1∼2.5%, sevoflurane, propofol 2 mg/kg/h, remifentanil 0.15 *μ*g/kg/min, and vecuronium 0.02∼0.03 mg/kg(injected once every 40 minutes) were used to maintain anesthesia.During the operation, the alveolar gas concentration of sevoflurane was monitored and the bispectral index (BIS) value was maintained at 40–60.

**Figure 1:**
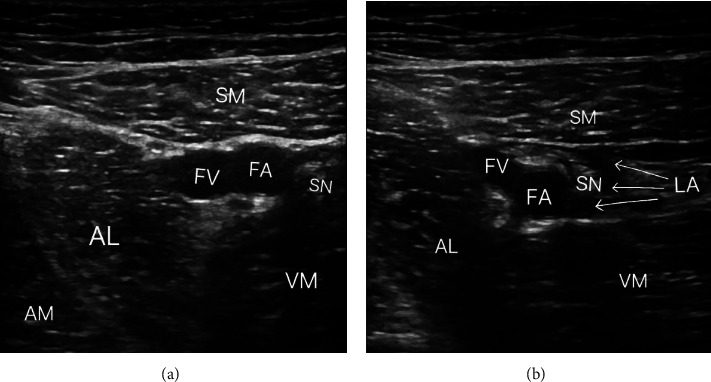
Ultrasound images (a) before and (b) after ACB. Abbreviations: SM, sartorius muscle; AL, adductor longus; AM, adductor magnus; VM, vastus medialis; FA, femoral artery; FV, femoral vein; SN, saphenous nerve; LA, local anesthetic.

Intravenous injection of 1.0 g of tranexamic acid was administered when loosening the tourniquet during the surgery, and patients were administered 0.5 g of tranexamic acid twice a day after surgery. All ACBs of the affected limb were performed after recovery of the patient and removal of the endotracheal tube in the postanesthesia care unit. The patient was placed in a supine position with the thigh and calf on the operative side mildly externally rotated, and the thigh was routinely disinfected and toweled. The probe (HFL38x/13–6MHzTransducer, SonoSite Inc., Bothell, WA, USA) was transversely placed laterally at the midpoint of the line between the patella and the anterior superior iliac spine, and the femoral artery, sartorius, adductor longus, and vastus medialis were located first. When the femoral artery is below the sartorius, the triangular lumen surrounded by the sartorius, vastus medialis, and adductor longus is the adductor canal, while the area where the medial edges of the sartorius and adductor longus intersect is the proximal of the adductor canal (Figure 1(a)). The hyperechoic structure on the lateral side of the femoral artery is the saphenous nerve. Using the in-plane technique, the needle is inserted advanced toward the femoral artery and from the lateral side to the medial side, with the tip of the needle placed beside the saphenous nerve. 20 ml of 0.33% ropivacaine (LBKL, AstraZeneca AB, Sweden) plus 1 mg butorphanol (Jiangsu Hengrui Medicine Co., Ltd, Lianyungang, Jiangsu, China) was injected in Group BR, and 20 ml of 0.33% ropivacaine plus 1 ml normal saline was injected in Group R. The drug was spread evenly along the perimeter of the saphenous nerve under an ultrasound image to ensure a complete block (Figure 1(b)). The successful block was defined as the disappearance of sensation in an innervation area of the saphenous nerve by the pinprick test.

### 2.5. Postoperative Management

The ice compressed around the patient's incision after surgery and PCIA (patient-controlled intravenous analgesia) was used to relieve pain postoperatively in all patients. PCIA formulation was as follows: dose 80 ml, flurbiprofen ester 100 mg, and tramadol hydrochloride injection 800 mg. PCIA electronic pump parameters were basal rate,1 ml; initial dose,1 ml; PCA dose, 2 ml; and lockout, 15 min. When the patient cannot tolerate pain or the pain score at rest is higher than 6 points, flurbiprofen ester (50 mg) is intravenously administered as rescue analgesia. The patient started physical function training of the knee joint 2 hours after the operation, and got out of bed as soon as possible for activities.

### 2.6. Data Collection

The primary outcomes were the duration of the sensory block and the visual analogue scale (VAS) of pain at rest and with activity (the patient that remained in the prone position with the thigh on the side of the operation was immobilized, and the knee joint was at 45° to the bed surface) at 4, 8, 12, 24, and 48 h after surgery, where a score of 0 represents no pain and 10 indicates worst pain. The duration of the sensory block was defined as the time from the onset of the block to its resolution as demonstrated by pinprick testing. We compared the patient's sensation on the block side with the normal side every hour after ACB, and the point of sensory block resolution was defined as the time point at which the sensation became equal on both sides. The secondary outcomes included the number of PCIA attempts, the time to first pressing, and the rescue analgesic rate. Other outcomes included evaluating knee functional recovery, which involved the knee active range of motion (ROM, use a goniometer to measure the angle between the calf and the bed surface when the patient keeps prone position and the knee is in the maximum flexion position), quadriceps strength (0 represents worst strength and 5 indicates best strength), the time to first mobilization, functional KSS (Knee Society Score, evaluating the ability of walking and climbing), and the duration of postoperative hospital stay. Furthermore, the occurrences of complications including PONV (postoperative nausea, and vomiting), pruritus, constipation, somnolence, uroschesis, and respiratory depression were also recorded after surgery.

### 2.7. Statistical Analyses

The sample size was determined by the nerve block duration. In the preliminary experiment, 10 patients were included in each of our two groups, and the duration of the nerve block in the two groups were, respectively, 17.01 ± 3.26 h and 14.62 ± 2.34 h. Assuming a power of 90% and *α* = 0.05 (2-tailed), the sample size calculated by PASS 15 software was 30 patients in each group. Finally, with an anticipated 20% dropout rate, we chose 40 people in each group to prevent dropouts.

All statistical analyses were performed using IBM SPSS 23.0 (IBM Corporation, Armonk, NY, USA). Normally distributed continuous data were expressed as a mean ± standard deviation (‾*x* ± *s*), and group comparisons were made using the group *t*-test. The non-normally distributed measures were expressed as a median (M) and interquartile range (IQR), and the Mann–Whitney *U* test was used for comparison between groups. Categorical data are presented as cases (%), and the *χ*^2^ test was used for comparison between groups. *p* < 0.05 was considered statistically significant.

## 3. Results

### 3.1. Patients

**Figure 2:**
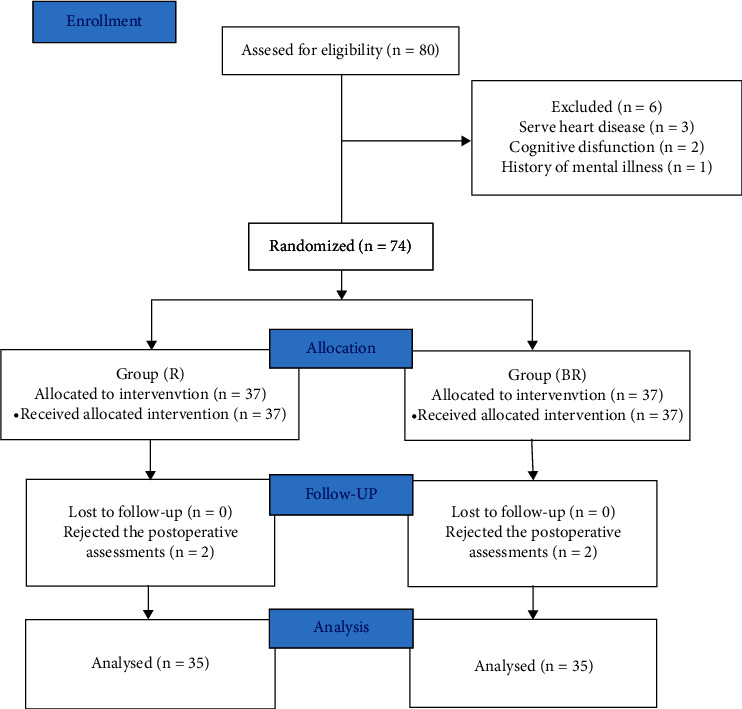
The flowchart.

The study flow diagram is presented in Figure 2. A total of 80 patients were screened initially, and 74 patients were enrolled in the study. The remaining eligible patients were randomized into two groups, 37 in the BR group and 37 in the R group. However, data from only 70 patients were analysed, and four people rejected the postoperative assessments. There were no significant differences between two groups in baseline characteristics before surgery, including age, gender, BMI, ASA status, preoperative pain scores, quadriceps strength, knee ROM, KSS function score, the time of the tourniquet, and operation time (Table 1).

### 3.2. Primary Outcomes and Secondary Outcomes

**Figure 3:**
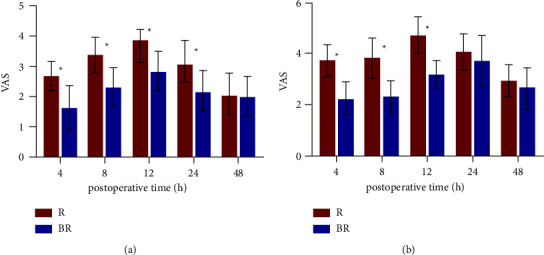
VAS pain scores at rest during 48 hours after operation. The results were represented by the bar graph. ^*∗*^*P* < 0.05 compared with Group R. (a) Incisional pain at rest. (b) Incisional pain with activity. Abbreviation: VAS, visual analogue scale.

Group BR had longer duration of the sensory block than Group R (18.42 ± 3.46 vs. 15.36 ± 2.29 h, *p* < 0.01). As shown in Table 2 and Figure 3(a), the VAS pain scores while resting at 4, 8, 12, and 24 hours postoperatively were significantly lower for the patients in the BR group than those in the R group (*p* < 0.01). Patients in the BR group had also significantly lower VAS scores for pain with activity at 4, 8, and 12 hours postoperatively when compared with the patients in the R group (*p* < 0.01) (Table 2 and Figure 3(b)). The number of PCIA attempts in the BR group was significantly less than that in the R group (4.5 ± 1.2 vs. 7.8 ± 1.5 times, *p* < 0.01), and the time of first pressing in the BR group was also later than that in the R group (20.31 ± 2.59 vs. 16.25 ± 2.31 h, *p* < 0.01) (Table 2). In addition, 7 patients in Group R (20.00%) required rescue analgesia for unbearable pain compared with 5 patients (14.29%) in the BR group (*p* > 0.05) (Table 2).

### 3.3. Other Outcomes

**Figure 4:**
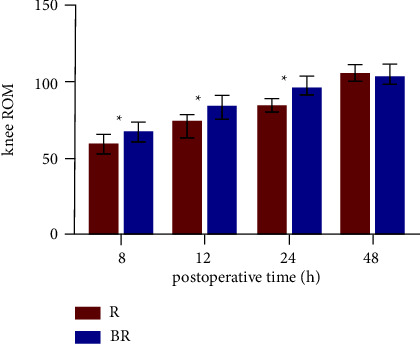
Knee ROM during 48 hours after operation. The results were represented by the bar graph. ^*∗*^*P* < 0.05 compared with Group R. Abbreviation: knee ROM, knee active range of motion.

The mean values of knee ROM were 68.37 ± 4.70° in the BR group and 59.21 ± 6.41° in the B group at 8 h after surgery, 85.67 ± 5.17° and 74.37 ± 4.68° at 12 h and 97.62 ± 5.43° and 84.18 ± 4.49° at 24 h in the BR group and R group, respectively (*p* < 0.05) (Table 3, Figure 4). However, there were no significant differences between two groups in quadriceps strength, the time to first mobilization, functional KSS at hospital discharge, and the duration of hospital stay (*p* > 0.05) (Table 3). PONV occurred in 10 (27.78%) patients in the BR group and 7 (20.00%) patients in the R group (*p* > 0.05). Two patients had pruritus in the R group. There were no occurrences of constipation, somnolence, uroschesis, and respiratory depression (Table 4).

## 4. Discussion

TKA is an effective solution to end-stage knee disorders, and the number of operations is increasing every year [1, 12]. However, as TKA requires osteotomy of the femoral and tibial articular surfaces, the operation can cause severe postoperative pain to patients [2, 13]. Furthermore, surgical stress-induced immune and inflammatory reactions increased the sensitivity of nerve fibers around the knee joint, leading to more pain [14]. ACB has become a mainstay for providing postoperative analgesia for TKA. Numerous clinical studies have shown that ACB can provide effective postoperative analgesia with a much smaller effect on quadriceps muscle strength and muscle power [15], facilitating the early removal of patients from bed and reducing the incidence of perioperative complications.

Currently, prolonging the duration and enhancing the analgesic effect of nerve blocks with local anesthetic adjuvants are one of the modalities of perioperative multimodal analgesia in TKA and are an attractive and technically simple analgesic strategy. Some studies have proved that dexmedetomidine and dexamethasone can significantly enhance the analgesic effect as local anesthetic adjuvants [16, 17], but the former is not the most desirable local anesthetic adjuvant because of its side effects such as bradycardia and the latter because of a slight increase in blood glucose [9, 18]. Opioid receptors are widely expressed in the central and peripheral nervous system as well as in non-neural tissues [19]. Local application of exogenous opioid agonists can activate peripheral opioid receptors in inflamed tissues, resulting in an effective analgesic effect. This analgesic effect avoids opioid receptors in the central nervous system and therefore is less likely to cause side effects such as respiratory depression, mental confusion, altered consciousness, or addiction [20].

Butorphanol is a highly selective opioid agonist-antagonist for *κ* receptors, with an affinity of 25 : 4:1 for *κ*, *μ,* and *δ* receptors, respectively, with a dose-dependent and capping effect on *κ* receptors, and a dual agonist and antagonist effect on *μ* receptors, which mediate many opioid adverse reactions [21, 22]. The features enable butorphanol to provide good analgesia with a low incidence of adverse effects. Clinically, butorphanol has been proven to be an effective adjuvant for epidural anesthesia and brachial plexus blocks. Bharti and Chari [11] concluded that addition of two mg of butorphanol to 0.125% of epidural bupivacaine resulted in rapid onset and longer duration of analgesia than did butorphanol alone. Kumari et al. [23] showed that butorphanol (2 mg) as an adjuvant to levobupivacaine in the supraclavicular block hastens the onset and prolongs the duration of the block. However, in a dose study of a combination of bupivacaine with 2 and 1 mg of butorphanol in brachial plexus nerve blocks, Bharathi et al. [10] reported significantly faster onset time (8.04 ± 0.65 min) and greater duration of analgesia (643.55 ± 131.6 min) with the addition of 2 mg of butorphanol to 0.25% bupivacaine as compared with 1 mg bupivacaine (onset time = 12.57 ± 3.5 min, duration = 511.73 ± 128.6 min). The incidence of nausea, vomiting, and pruritus was observed in more number of patients in the former. Hence, we chose 1 mg butorphanol for the use in ACB. Although the volume of a local anesthetic was 29 ml in the study by Bharathi B, our study showed that the duration of analgesia of ropivacaine with or without butorphanol (18.42 ± 3.46 vs. 15.36 ± 2.29 h) was longer in adductor canal blocks than that in brachial plexus blocks, and it may be due to the adductor canal being in a relatively closed space, which could limit the spread of a local anesthetic to the surrounding area. Based on previous research, the minimum clinically important difference (MCID) in acute postoperative pain measured with the VAS pain score was 1.0, which means less consumption of postoperative analgesics [24, 25], and a mean VAS difference of 1.3 or greater has been considered clinically significant [26]. At 4, 8, and 12 hours after surgery, the VAS difference between the two groups was statistically significant, and both reached MCID. In addition, the mean difference of pain scores with activity at 4, 8, and 12 hours after surgery was higher than 1.5. This indicates that butorphanol plus ropivacaine in ACB provides better pain control in the early time after TKA, especially in motion. There were fewer numbers of PCIA attempts (4.5 ± 1.2 vs. 7.8 ± 1.5) and longer time (16.25 ± 2.13 vs. 20.31 ± 2.59 hours) to first press postoperatively, suggesting that butorphanol combined with ropivacaine ACB could reduce postoperative opioid consumption. However, there was no significant difference in the rate of rescue analgesia between the two groups, which may be due to the fact that the duration of analgesia did not exceed 24 hours, as we observed that most rescue analgesia occurred on the second postoperative day. Regarding the mechanism by which butorphanol prolongs the analgesic duration of nerve blocks and enhances the analgesic effect, some scholars believe that it enhances the anti-injurious effects of local anesthetics by closing influx membrane calcium channels and opening membrane potassium channels to cause hyperpolarization of the cell membrane potential and suppression of action potential transmission of ascending pain pathways through G protein-coupled receptors [27]. Furthermore, some studies [28] found that butorphanol could alleviate formalin-induced inflammation of the temporomandibular joint, and Vavhon and Moreau [29] also found that subcutaneous injection of butorphanol in experimental rats could effectively reduce carrageenan-induced paw edema, which suggests that butorphanol also has some anti-inflammatory effects.

The rapid recovery of patients after TKA depends on getting out of bed as early as possible for appropriate functional knee exercises [15]. In this study, although 1 mg butorphanol compounded with 0.33% ropivacaine in 20 ml of ACB failed to shorten the time to first mobilization, it significantly improved knee mobility at 8, 12, and 24 h after surgery and had no effect on quadriceps muscle strength, indicating that butorphanol compounded with ropivacaine helped patients recover knee function. Although the difference in VAS scores with activity between the two states at 24 h postoperatively was not statistically significant, there was a noticeable difference in knee ROM between the two groups at this time point, suggesting that knee mobility is not only related to the degree of pain, but increased knee mobility at an earlier time point and earlier movement out of bed would also help improve joint stiffness [30] and reduce inflammatory adhesions, which in turn would be beneficial in the long term for the recovery of knee function.

The occurrence of PONV can prolong the length of stay in the postanesthesia care unit (PACU) as well as the length of hospital stay [31]. In this study, there was no statistical difference in the incidence of PONV between the two groups, and two patients had pruritus in the R group, but the incidence of pruritus in the two groups was not statistically significant. We did not observe constipation, somnolence, uroschesis, and respiratory depression in any patient, suggesting that butorphanol as a local anesthetic adjuvant did not increase the incidence of these complications. In fact, butorphanol has been shown to reduce the incidence and severity of pruritus after cesarean section without affecting the quality of postoperative analgesia, and the mechanism lies in the antagonism of butorphanol on *μ* receptors [32]. A pharmacological study also found that butorphanol inhibited histamine-induced pruritus, reducing itch by 35%, which was associated with the activation of the basal nucleus of Meynert, the nucleus accumben, the septal nuclei, and the adjacent cingulate gyrus area [33].

The study has some limitations. First, the dose of butorphanol was single, and it was not shown how different doses of butorphanol affect research results. Second, we did not measure any inflammatory factor associated with pain severity, and combining laboratory tests in the future can confirm the conclusion better.

## 5. Conclusions

The application of 1 mg butorphanol compounded with ropivacaine for ACB after TKA can prolong the duration of sensory blocks, effectively relieve patients' early postoperative pain, reduce the number of PCIA attempts, prolong the time to first press, and encourage early improvement in patients' knee function. Further research is needed to see whether the most appropriate dose of butorphanol combined with ropivacaine for ACB affects postoperative analgesia of TKA.

## Figures and Tables

**Table 1:** Patient characteristics.

	Group R (*n* = 35)	Group BR (*n* = 35)	*P* value
Age (years)	66.4 ± 7.2	66.6 ± 7.9	0.925
Gender (male/female)	10/25	7/28	0.403
BMI (kg/m^2^)	25.0 ± 3.3	25.4 ± 2.8	0.514
ASA (II/III)	20/15	22/13	0.626
VAS pain score (prior to surgery)	4.17 ± 1.25	3.86 ± 1.14	0.275
Knee ROM (prior to surgery)	119.98 ± 6.55	119.33 ± 5.89	0.665
KSS function score	22.6 ± 3.7	23.5 ± 2.5	0.205
Quadriceps strength	5.0 (4.0, 5.0)	5.0 (4.0, 5.0)	0.207
Duration of the tourniquet (min)	61.6 ± 9.6	65.6 ± 10.2	0.104
Duration of the operation (min)	95.7 ± 10.5	91.5 ± 9.7	0.088

*Note.* Values are expressed as a mean ± SD, median (IQR), or number. Abbreviations: BMI, body mass index; ASA, American Society of Anesthesiologists; VAS, visual analogue scale; ROM, range of motion; KSS, Knee Society Score.

**Table 2:** Postoperative pain assessment.

	Group R (*n* = 35)	Group BR (*n* = 35)	*P* value
Duration of sensory blocks (h)	15.36 ± 2.29	18.42 ± 3.46	0.001
VAS at rest			
4 h	2.69 ± 0.47	1.63 ± 0.73	0.001
8 h	3.37 ± 0.60	2.29 ± 0.67	0.001
12 h	3.86 ± 0.36	2.83 ± 0.66	0.001
24 h	3.06 ± 0.80	2.17 ± 0.66	0.001
48 h	2.03 ± 0.74	1.97 ± 0.71	0.707
VAS with activity			
4 h	3.77 ± 0.60	2.26 ± 0.66	0.001
8 h	3.86 ± 0.77	2.31 ± 0.63	0.001
12 h	4.74 ± 0.70	3.20 ± 0.53	0.001
24 h	4.09 ± 0.70	3.74 ± 0.98	0.097
48 h	2.97 ± 0.61	2.71 ± 0.75	0.122
Number of PCIA attempts	7.8 ± 1.5	4.5 ± 1.2	0.001
Time of first press (h)	16.25 ± 2.13	20.31 ± 2.59	0.001
Rescue analgesic cases (*n*, (%))	7 (20.0%)	5 (14.3%)	0.526

*Note.* Values are expressed as a mean ± SD or number (%). Abbreviations: 4 h, 4 hours after surgery; VAS, visual analog score; PCIA, patient-controlled intravenous analgesia.

**Table 3:** Postoperative knee functional rehabilitation.

	Group R (*n* = 35)	Group BR (*n* = 35)	*P*-value
Quadriceps strength			
8 h	3.0 (3.0, 3.0)	3.0 (3.0, 4.0)	0.358
12 h	3.0 (3.0, 4.0)	4.0 (3.0, 4.0)	0.435
24 h	5.0 (4.0, 5.0)	5.0 (4.0, 5.0)	0.223
48 h	5.0 (5.0, 5.0)	5.0 (5.0, 5.0)	0.090
Knee ROM (degrees)			
8 h	59.21 ± 6.41	68.37 ± 4.70	0.001
12 h	74.37 ± 4.68	85.67 ± 5.17	0.001
24 h	84.18 ± 4.49	97.62 ± 5.43	0.001
48 h	104.65 ± 6.01	106.45 ± 4.54	0.160
Time to first mobilization (h)	17.01 ± 4.83	16.20 ± 4.06	0.440
KSS function score at discharge	33.4 ± 4.7	34.6 ± 4.3	0.271
Postoperative hospital stay (h)	70.80 ± 11.56	72.09 ± 10.13	0.619

*Note.* Values are expressed as a mean ± SD or median (IQR). Abbreviations: ROM, range of motion; KSS, Knee Society Score.

**Table 4:** Postoperative complications.

	Group R (*n* = 35)	Group BR (*n* = 35)	*P* value
PONV	10 (28.6)	7 (20.0)	0.403
Pruritus	2 (6.1)	0 (0.0)	0.473
Constipation	0 (0.0)	0 (0.0)	>0.05
Somnolence	0 (0.0)	0 (0.0)	>0.05
Uroschesis	0 (0.0)	0 (0.0)	>0.05
Respiratory depression	0 (0.0)	0 (0.0)	>0.05

*Note.* Values are expressed as a number (%). Abbreviations: PONV, postoperative nausea and vomiting.

## Data Availability

The datasets generated and/or analysed during the current study are not publicly available due to limitations of ethical approval involving the patient data and anonymity but are available from the corresponding author on reasonable request.

## Abbreviations

ACB: Adductor canal block
TKA: Total knee arthroplasty
VAS: Visual analogue scale
PCIA: Patient-controlled intravenous analgesia
ROM: Range of motion
KSS: Knee Society Score
ASA: American Society of Anesthesiology
BMI: Body mass index
PACU: Postanesthesia care unit
ERAS: Enhanced recovery after surgery
ECG: Electrocardiogram
SpO_2_: Blood oxygen saturation
IBP: Invasive blood pressure
PETCO_2_: Partial pressure of end respiratory carbon dioxide
BIS: Bispectral index
SM: Sartorius muscle
AL: Adductor longus
AM: Adductor magnus
VM: Vastus medialis
FA: Femoral artery
FV: Femoral vein
SN: Saphenous nerve
LA: Local anesthetic.
